# Outcomes of Difficult-to-Treat Ulcerative Colitis: Impact of Primary Nonresponse vs Secondary Loss of Response to Prior Therapies: A Retrospective Cohort Study

**DOI:** 10.1016/j.gastha.2026.100949

**Published:** 2026-04-03

**Authors:** Terry Lou, Yuchen Qi, Melissa Kirkpatrick, Brigid S. Boland, Siddharth Singh

**Affiliations:** 1 Department of Medicine, University of California San Diego, La Jolla, California; 2 Division of Biostatistics and Bioinformatics, Herbert Wertheim School of Public Health, University of California San Diego, La Jolla, California; 3 Department of Pharmacy, University of California San Diego, La Jolla, California; 4 Division of Gastroenterology, Department of Medicine, University of California San Diego, La Jolla, California; 5 Division of Gastroenterology and Hepatology, Department of Medicine, Mayo Clinic Arizona, Scottsdale, Arizona

Treatment options for inflammatory bowel diseases (IBD) have expanded significantly in the last decade; however, a substantial proportion of patients does not attain or maintain disease remission. “Difficult-to-treat” (DTT) IBD has been defined as failure to respond to 2 advanced therapies with different mechanisms of action, with 19% patients treated with advanced therapies meeting criteria in a referral center study.^1^^,^^2^ It is unclear whether outcomes differ based on reasons for failure of prior advanced therapies—primary nonresponse (PNR; defined as lack of response to induction therapy despite attempted optimization, which often reflects mechanistic failure) vs secondary loss of response (LOR; defined as loss of response during maintenance therapy after initial clinical improvement, either due to immunogenicity or immunologic escape). We examined outcomes of DTT ulcerative colitis (UC) based on reasons for discontinuation of prior advanced therapies.

We conducted a single-center retrospective cohort study of adult patients with DTT-UC (IRB# 807843) starting a third line advanced therapy after failure of at least 2 classes of advanced therapies between 2016 and 2023 with at least 1-year follow-up after initiation of third line therapy. Patients were classified as PNR to both prior advanced therapy classes (PNR/PNR), PNR to 1 prior advanced therapy and LOR to another prior advanced therapy (PNR/LOR or LOR/PNR), or LOR to both prior advanced therapy classes (LOR/LOR); a few patients who discontinued therapy due to intolerance were grouped with LOR (see Supplementary Material for approach to classification). Our primary outcome was time to treatment failure of third line advanced therapy, defined as a composite of UC-related hospitalizations, colectomy, new corticosteroid use [occurring >8 weeks after initiation of advanced therapy], and discontinuation of third line advanced therapy). We conducted Cox proportional hazard analysis, adjusting for age, class of third line advanced therapy, endoscopic severity, albumin, and concomitant corticosteroid use.

We included 104 patients (42 ± 15 years; 45% female, 74% Caucasian, 3% African American, 4% Asian, and 19% classified as mixed race or other) with DTT-UC followed over 3.0 ± 2.1 years. The following therapies were initiated as third line index therapy: janus kinase inhibitors (tofacitinib [25%], upadacitinib [16%]), ustekinumab (25%), tumor necrosis factor (TNF) antagonists (20%), and vedolizumab (14%). TNF antagonists (75%, including 45% infliximab) and vedolizumab (23%) were the most common 1st line therapies that patients failed, and vedolizumab (47%) and TNF antagonists (36%) were the most common second line therapies that patients failed. Overall, 23 patients (22%) experienced PNR/PNR to both first line advanced therapies, 39 patients (37.5%) had PNR/LOR or LOR/PNR, and 42 patients (40.5%) experienced LOR/LOR. On follow-up over 3 years, 81% patients experienced treatment failure, including 33% patients with IBD-related hospitalization, 12% undergoing colectomy, and 71% requiring corticosteroids. Approximately 72% discontinued their third line advanced therapy within 12.5 ± 12.4 months, and 65% were started on fourth line advanced therapy (janus kinase inhibitors [53%], ustekinumab [29%], TNF antagonists [10%], vedolizumab [5%], and IL23p19 antagonists [3%]). Only 24% patients achieved endoscopic remission within 1 year. Reason for discontinuation of prior advanced therapies was not associated with risk of treatment of failure, whether examined as PNR/PNR vs PNR/LOR or LOR/PNR vs LOR/LOR (*P* = .91), PNR/PNR vs PNR to neither or 1 prior advanced therapy (*P* = .87), or PNR to any prior advanced therapy vs LOR/LOR (*P* = .67) (Figure A–C). Adjusting for covariates, severe endoscopic activity (odds ratio [OR], 2.93; *P* = .037), concomitant prednisone use (OR 2.88; *P* = .002), and younger age (OR 0.98; *P* = .039) at initiation of third line advanced therapy were associated with higher risk of treatment failure.

**Figure:**
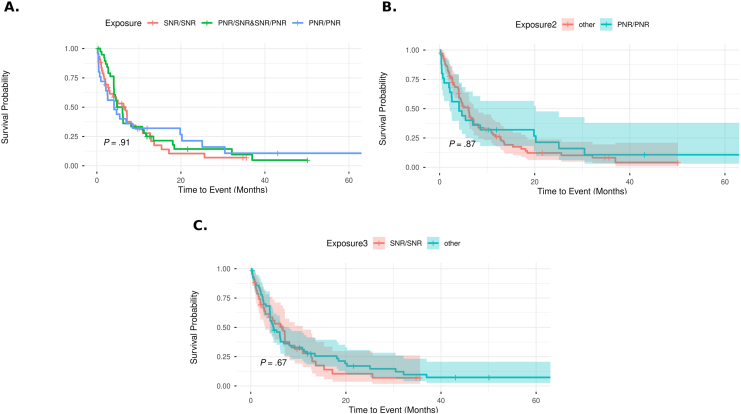
Survival free of treatment failure in patients with difficult-to-treat ulcerative colitis treated with third-line advanced therapy, based on reasons for discontinuation of prior 2 classes of advanced therapies. (A) PNR/PNR vs PNR/LOR or LOR/PNR vs LOR/LOR. (B) PNR/PNR vs any other combination (PNR/LOR or LOR/PNR or LOR/LOR). (C) LOR/LOR vs any other combination (PNR/LOR or LOR/PNR or PNR/PNR). LOR or SNR, loss of response or secondary nonresponse; PNR, primary nonresponse.

In a prior meta-analysis, George and colleagues^3^ observed that patients who experience PNR to TNF antagonists have lower likelihood of achieving remission with second-line therapy compared with patients who experience secondary LOR. In a subsequent patient-level synthesis of clinical trials of ustekinumab and vedolizumab in patients with UC, there was no difference in likelihood of response to induction therapy in patients who discontinued prior TNF antagonist due to PNR vs secondary LOR, though both of these groups of patients had lower likelihood of response compared with patients who discontinued their first biologic due to intolerance.^4^ In clinical trials conducted around 2000s–2010s, when therapeutic drug monitoring and treatment optimization was infrequent, pharmacokinetic failure was likely the primary driver of biologic failure in PNR and LOR. In contrast, in our referral center practice, therapeutic drug monitoring with treatment optimization is frequently performed either proactively or in patients with suboptimal response. Consequently, most patients who experienced PNR and LOR in our cohort were more likely to have experienced mechanistic failure, rather than pharmacokinetic failure, leading to similarly poor outcomes. Krishna and colleagues also observed that outcomes with second-line advanced therapies were comparable in patients who experienced PNR vs secondary LOR to first-line therapy.^5^

There have been limited studies of third line therapies in patients with DTT-IBD. In a 2-center retrospective cohort study of 430 patients meeting criteria for DTT-IBD, only 26% patients achieved endoscopic remission compared with 62% patients who did not have DTT-IBD.^2^ Treatment persistence of third and subsequent lines of therapy was significantly lower compared with prior lines of therapy.

While our study provides meaningful clinical insights, there are certain limitations to acknowledge. First, the number of patients with DTT-IBD who could be categorized into PNR/PNR, PNR/LOR, LOR/PNR, or LOR/LOR was small and may have been underpowered to detect meaningful differences. We did not capture patient-reported outcomes and biochemical remission endpoints routinely but relied on hard outcomes like surgery, hospitalization, and corticosteroid use. Second, we did not routinely capture data on drug concentrations and immunogenicity, making it challenging to explicitly categorize treatment failure as mechanistic or pharmacokinetic. Nevertheless, therapeutic drug monitoring and treatment optimization is part of routine clinical practice at our referral center. Classification of PNR and LOR was also based on retrospective interpretation of clinical notes, which may lead to misclassification. Third, with availability of newer therapies, practice patterns have evolved. Upadacitinib, which is one of the most efficacious medications for treating refractory UC, has only recently been introduced, and outcomes of DTT-UC patients are likely to improve with widespread utilization of this agent.^6^, ^7^, ^8^

In summary, 80% patients with DTT-UC with prior failure to 2 classes of advanced therapies will experience treatment failure with third line advanced therapy. Outcomes are similar regardless of PNR or LOR to one or both prior advanced therapies in these patients. Future studies characterizing mechanistic vs pharmacokinetic drivers of treatment failure are warranted to more optimally guide precision medicine in DTT-IBD.

## Supplementary Materials

Supplementary Material

Extended PDF
