# Supplementary material for: Outcomes of Difficult-to-Treat Ulcerative Colitis: Impact of Primary Nonresponse vs Secondary Loss of Response to Prior Therapies: A Retrospective Cohort Study
Source: Gastro Hep Adv. 2026 Apr 3;5(6):100949. doi: 10.1016/j.gastha.2026.100949 (PMC13196064; doi:10.1016/j.gastha.2026.100949)
Supplement: Supplementary Material [file mmc1.pdf]

## METHODS

Patients were classified as primary non-response (PNR) or secondary loss of response (LOR) using a hierarchical approach. If clinical notes (written by gastroenterologists specializing in the care of patients with IBD) clearly mentioned primary non-response vs. secondary LOR, then we relied on notes. When this was not clearly mentioned, we relied on language in the notes. Patients were classified as PNR if the notes suggested the patient did not have clinical response to induction therapy, and the medication was typically discontinued within 3-6 months of initiation. Patients were classified as secondary LOR if it appeared the patient responded initially, and then subsequently lost response to therapy with discontinuation of therapy within 6-18 months.
